# Central nervous system infection caused by *Mycobacterium houstonense*: A case report

**DOI:** 10.3389/fneur.2022.908086

**Published:** 2022-09-01

**Authors:** LiXia Wang, FaPing Wang, Chuan Yang, FengMing Luo

**Affiliations:** ^1^Department of Respiratory and Critical Care Medicine, West China Hospital, Sichuan University, Chengdu, China; ^2^Laboratory of Pulmonary Immunology and Inflammation, Frontiers Science Center for Disease-related Molecular Network, Sichuan University, Chengdu, China

**Keywords:** *Mycobacteria houstonense*, *Mycobacterium fortuitum* group, rapidly growing mycobacteria, central nervous system infection, next generation sequencing

## Abstract

**Background:**

*Mycobacterium houstonense* is a rapidly growing mycobacterium (RGM) that belongs to the unnamed third biovariant complex of the *Mycobacterium fortuitum* group, which is rarely responsible for human infection. Approximately 76% of infections caused by the *M. fortuitum* group occur after open fractures or skin, soft tissue, bone, or puncture wounds. To date, only a few cases of human infectious disease caused b*y M. houstonense* have been reported worldwide.

**Case presentation:**

We present a case of a 26-year-old man with a central nervous system (CNS) infection caused by *M. houstonense*. The patient was transferred to our hospital because of headaches and muscle strength changes. One month prior to presentation at our hospital, the patient was diagnosed with tuberculous meningitis at the other two hospitals, but his condition did not improve after anti-tuberculous treatment, antibiotics, and anti-viral treatment before admission to our hospital. Lumbar puncture was performed at both previous hospitals, as well as at our hospital; the results consistently indicated high cerebrospinal fluid (CSF) opening pressure. *M. houstonense* was detected in the CSF of the second hospital's lumbar puncture by metagenomic next-generation sequencing (mNGS) but was not identified at our hospital. The patient was discharged from our hospital after receiving non-tuberculous mycobacterium (NTM) treatment for 1 month according to the Chinese NTM guidelines. However, the patient died 20 days after discharge.

**Conclusion:**

Since it is difficult to identify *M. houstonense*, this is the first case of human CNS infection caused by *M. houstonense* in China. This case may be considered by neurologists and infectious physicians when CNS infection does not respond to conventional treatment, especially in the uncommon type of NTM.

## Introduction

*Mycobacteria houstonense* is an acid-fast, gram-positive, sorbitol-positive, pleomorphic bacillus. Long filamentous forms are often observed, but the organisms have no spores or capsules (1). Rapidly growing mycobacteria (RGM) account for half of the known mycobacterial species and are divided into six major groups, including *Mycobacterium fortuitum* group, *Mycobacteria chelonae/Mycobacteria abscessus* complex, *Mycobacteria smegmatis* group, *Mycobacteria mucogenicum* group, *Mycobacteria mageritense/Mycobacteria wolinskyi* complex, and pigmented RGM (2). Members of the *M. fortuitum* group can cause disease in fish and other animals including humans. This group includes *M. fortuitum, Mycobacteria peregrinum*, and the unnamed third biovariant complex (*Mycobacteria senegalense, Mycobacteria porcinum, Mycobacteria houstonense, Mycobacteria neworleansense, Mycobacteria boenickei, Mycobacteria conceptionense, Mycobacteria septicum*, and *Mycobacteria alvei*) (3, 4). Here, we report a case of central nervous system (CNS) infection in China. *M. houstonense* was first isolated from the facial wound of a patient who lived in Houston, Texas, that's how *M. houstonense* got its name (1). To the best of our knowledge, this is the first report of human CNS infection caused by *M. houstonense* in China.

## Case presentation

A 26-year-old man was transferred to our hospital with complaints of dizziness, headache, insomnia for 1 month, and exacerbated limb weakness for half a month. One month ago, the patient first presented to the local hospital for these symptoms. Enhanced MRI of the brain showed a negative result while enhanced MRI of the cervical spine showed slight thickening of the cervical spinal cord. Upon lumbar puncture, the opening pressure was found to be more than 330 mmH_2_O and the cerebrospinal fluid (CSF) was transparent yellow, with a glucose (GLU) level of 3.8 mmol/L (reference range, 2.5–4.4 mmol/L), a microprotein level of 3.4 g/L (reference range, 0.15–0.45 g/L), and a chloride ion level of 112 mmol/L(reference range, 120–130 mmol/L). There were 50 nucleated cells per μl (reference range, 0–10), of which 95% were monocytes and 5% were coenocytes (Table 1). Considering the results of the CSF analysis (mononuclear-predominant CSF pleocytosis and a high protein level), the high open pressure, symptoms of intracranial hypertension, the inflammatory lesion on MRI findings, and the high incidence of tuberculosis in China [age-standardized incidence of tuberculosis, 54.18 per 100,000 population from 1990 to 2007 (5)], the patient was initially diagnosed with tuberculous meningitis and myelitis. He then received empirical antituberculosis therapy (a standard four-drug regimen of isoniazid, linezolid, moxifloxacin, and tigecycline). However, empirical treatment with anti-tuberculous drugs, antibiotics, antiviral drugs, and drugs to reduce the intracranial pressure (**Figure 4**), did not improve his condition. Thereafter, the patient was transferred to another hospital and, a lumbar puncture was repeated, the opening pressure was more than 330 mmH_2_O. The CSF was slightly yellow, with a GLU level of 5.35 mmol/L (reference range, 2.5–4.4 mmol/L), a microprotein level of 5.179 g/L (reference range, 0.08–0.43 g/L), a chloride ion level of 106.5 mmol/L (reference range, 120–130 mmol/L), there were 129 nucleated cells per microliter (reference range, 0–10), of which 94% were monocytes and 6% multinucleated cells (Table 1). The acid-fast staining, mycobacterium culture, Xpert MTB, and RT-PCR of the CSF samples were negative. *M. houstonense* was identified in the CSF by metagenomic next-generation sequencing (mNGS) in a company for gene sequencing named Smicere Diagnostics and the report is attached as a Supplementary material 1 which showed the reads of 264 (Supplementary material 1). The pathogen's data were aligned to the National Center for Biotechnology Information GenBank database and revealed sequence homology above 96.825 and 97.183% with *M. houstonense* (GenBank accession no. NZ_LT546208.1 and no. NZ_LT546207.1), respectively (Supplementary material 2). After 7 days, the CSF sample pathogen cultures in blood agar at 35°C from another lumbar puncture were negative. Despite empirical treatments with anti-tuberculous drugs (isoniazid, linezolid, moxifloxacin, and tigecycline) which are the same treatment in the previous hospital due to insufficient course (Figure 4), the patient's condition worsened (decreasing myodynamia of limbs and endorsed racing thoughts) and he was transferred to our hospital. This patient did not have any underlying diseases or past medical history, and he was employed in the field of information technology.

**Table 1:** CSF findings.

**Variable**	**Referrence range, The first hospital**	**The first hospital**	**Referrence range, The second hospital**	**The second hospital**			**Referrence range, Our hospital**	**Our hospital**					
Date		Dec 25, 2021		Dec 29, 2021	Jan 5, 2022	Jan 12,2022		Jan 14, 2022	Jan 21, 2022	Jan 24, 2022	Jan 28, 2022	Feb 7, 2022	Feb 14, 2022
Opening pressure (mmH_2_O)	80–180	>330	80–180	>330	>330	>330	80–180	150	>330	>330	>330	>330	>330
Appearance		Yellow		Light yellow	Light yellow	Light yellow		Yellow, slightly turbid	Light yellow, transparent	Light yellow, transparent	Light yellow, transparent	Yellow, transparent	Colorless, transparent
Nucleated cell count(cells/ul)	0–10	50		129	69	44	0–10	10	18	18	16	70	45
Proportion of mononuclear (%)		95		94	95	91		NA	NA	NA	NA	94	96
Microprotein (g/L)	0.15–0.45	3.400	0.08–0.43	5.179	4.964	4.827	0.15–0.45	11.480	9.160	7.740	6.560	6.500	7.150
CSF glucose (mmol/L)	2.5–4.4	3.80	2.5–4.4	5.35	3.53	4.26	2.5–4.4	5.04	4.05	4.24	4.41	2.74	4.24
Synchronous blood glucose (mmol/L)	3.9–5.9	NA	3.9–6.1	NA	NA	NA	3.9–5.9	8.42	5.51	6.37	7.00	5.57	8.72
CSF chloride ion (mmol/L)	120–130	112	120–130	106.5	107.5	96.8	120–130	108	104	101	110	112	105
Synchronous blood chloride ion (mmol/L)	99–110	NA	90–110	NA	NA	NA	99–110	98.6	91.8	94.8	96.7	100.8	100.1
IgG synthesis rate (mg/day)	0–5.81	NA	0–5.81	NA	NA	NA	0–5.81	NA	251.70	34.99	124.84	301.92	146.00
mNGS		NA		NA	*M. houstonense* was detected	NA		NA	Negative	Negative	NA	Negative	NA
Acid-fast staining		Negative		Negative	Negative	Negative		Negative	Negative	Negative	Negative	Negative	Negative
Mycobacterium culture		Negative		Negative	Negative	Negative		Negative	Negative	Negative	Negative	Negative	Negative
Xpert MTB		Negative		Negative	Negative	Negative		Negative	Negative	Negative	Negative	Negative	Negative
RT-PCR		Negative		Negative	Negative	Negative		Negative	Negative	Negative	Negative	Negative	Negative
Culture of *M. houstonense*		NA		NA	NA	Negative		NA	Negative	Negative	NA	Negative	NA

NA, not available.

**Figure 1:**
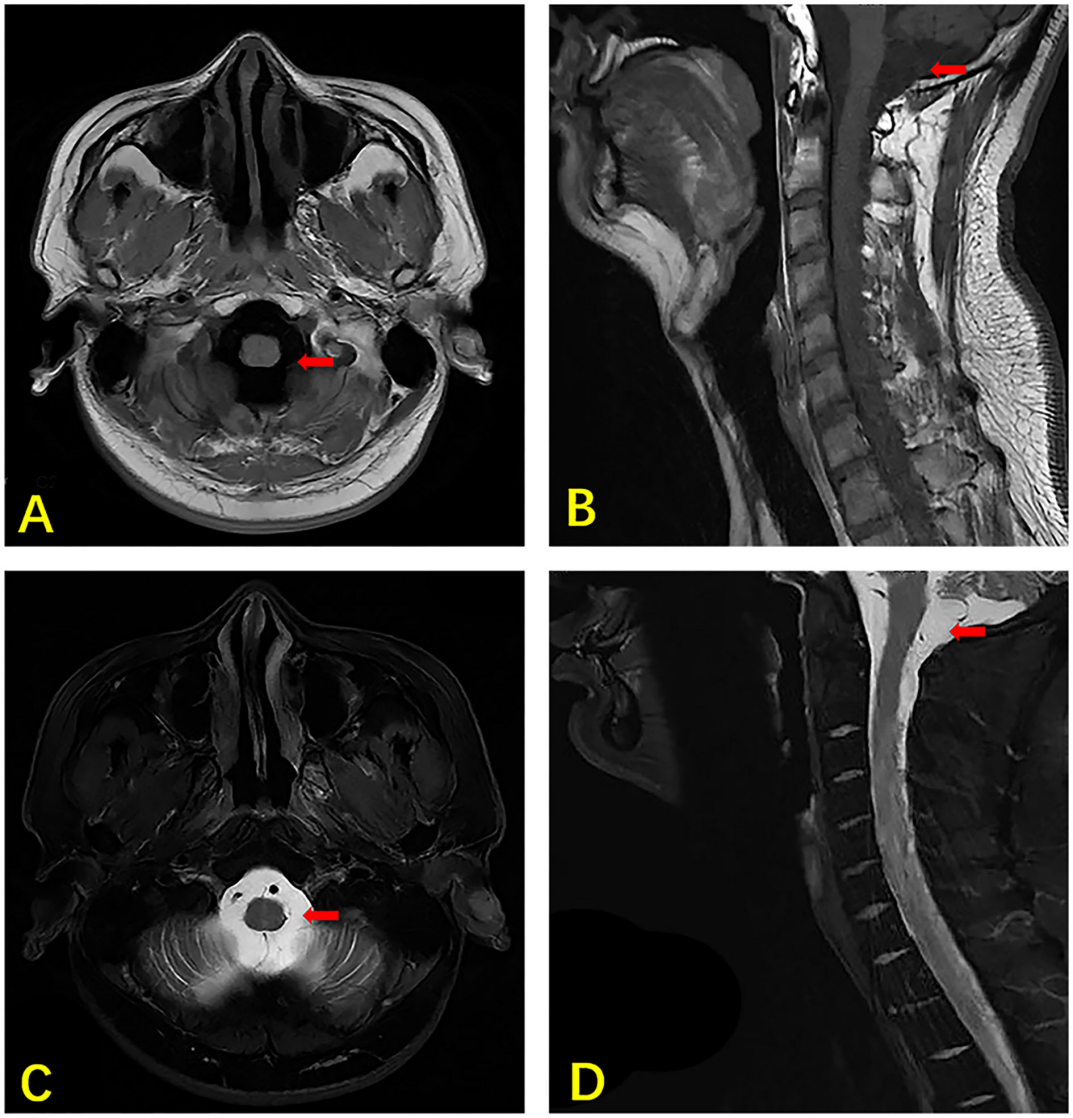
MRI of the brain and cervical vertebra of our case. The axial section T1 weighted image showing hydrocephalus (arrow) **(A)**. The sagittal section T1 weighted image showing hydrocephalus (arrow) **(B)**. The axial section T2 weighted image shows hydrocephalus (arrow) **(C)**. The sagittal section T2 weighted image showing hydrocephalus (arrow) **(D)**.

**Figure 2:**
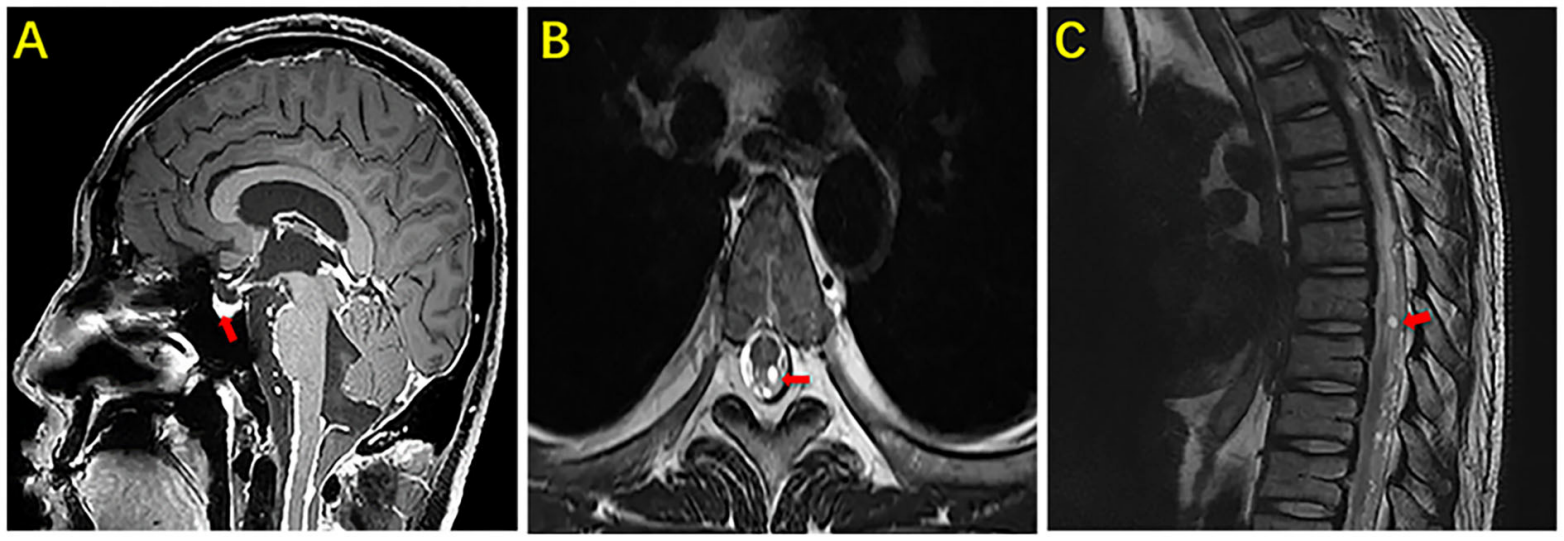
MRI of the brain and thoracic vertebra of our case. The sagittal section enhanced the image of the brain showing marked nodular enhancement under the pia mater (arrow) **(A)**. The axial section and sagittal section of the thoracic vertebra showing marked nodular enhancement in the spinal cord (arrow) **(B)**. The sagittal section of the thoracic vertebra showing marked nodular enhancement in the spinal cord (arrow) **(C)**.

**Figure 3:**
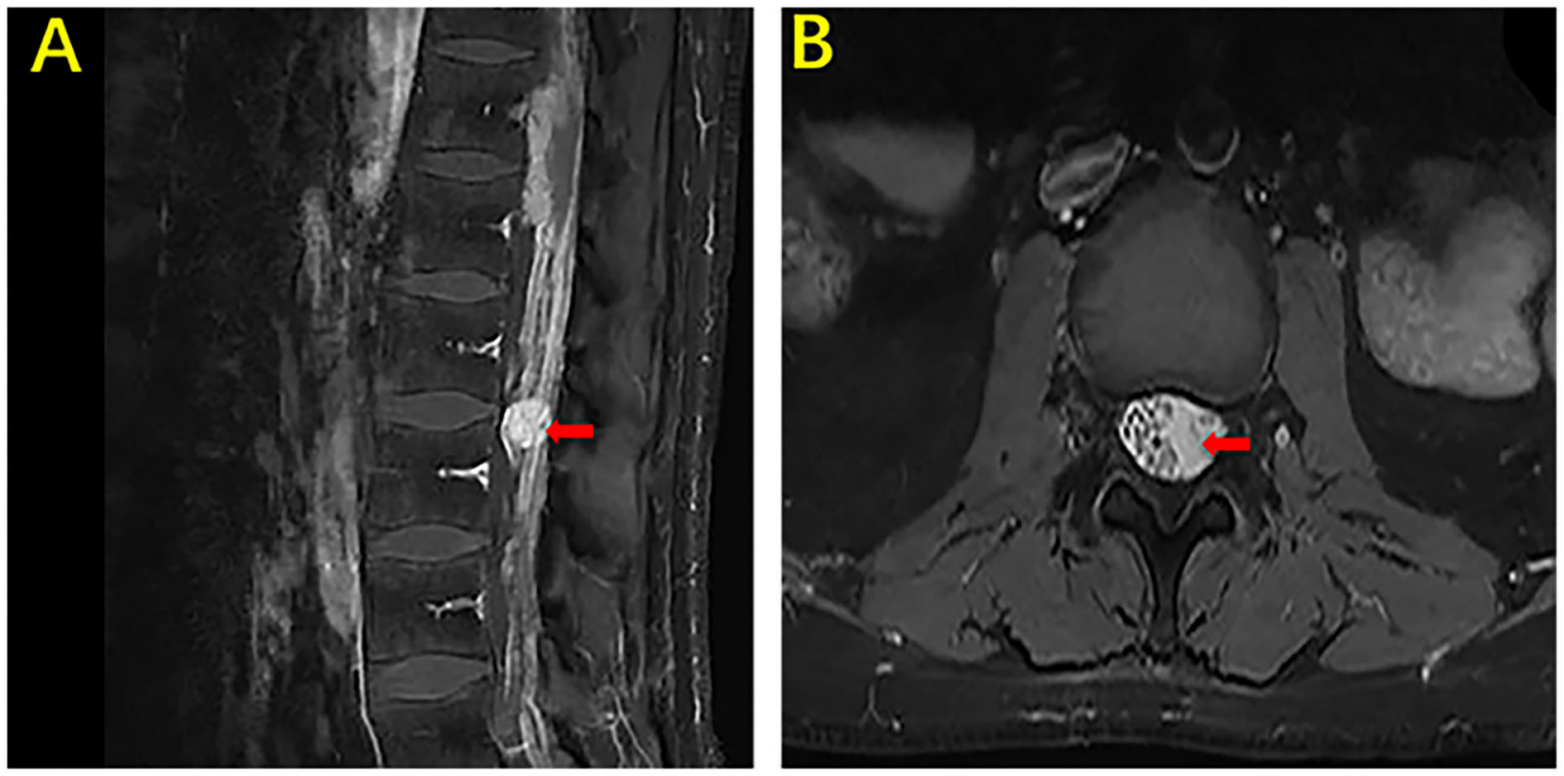
MRI of the brain and lumbar vertebra of our case. The sagittal section and axial section of the lumbar vertebra showing marked nodular enhancement in the spinal cord at the level of the second lumbar vertebra (arrow) **(A)**. The axial section of the lumbar vertebra showing marked nodular enhancement in the spinal cord at the same level in picture **(A)** (arrow) **(B)**.

After being admitted to our hospital, the patient's neurological examination showed the myodynamia of the left limb was grade 2, the right upper limb was grade 4, and less than grade 4. The muscle tension on the left side was decreased and that on the right side was normal. Poor abduction of the eyes and tongue deviation to the left was observed. The symmetry of the tendon reflexes in the extremities was weakened, and the craniocervical flexion test and bilateral Kerning sign were positive. The finger-to-nose test, heel-knee-shin test, Romberg test, and other ataxic tests could not be performed. The laboratory tests showed that there were 6.11 × 10^9^ erythrocytes per L, 312 × 10^9^ platelets per L, and 23.11 × 10^9^ leukocytes per L, of which 93.4% were neutrophils. His blood biochemistry was normal except for the raised glutamic-pyruvic transaminase level of 201 IU/ L (reference range, <40 IU/L) and glutamic oxalacetic transaminase of 53 IU/L (reference range, <35 international IU/L), and a mild reduced albumin level of 34.7 g/L (reference range, 40–55 g/L). His inflammatory biomarkers showed an elevated procalcitonin level of 4.03 n/ml (reference range, <0.046 ng/ml), C-reaction protein level of 15 mg/L (reference range, <5 mg/L), interleukin-6 level of 5.81 pg per ml (reference range, 0–7 pg per ml). His HIV antibody test was negative, but there were 502 CD3^+^ T cells per μl (reference range, 941–2,226), 269 CD4^+^ T cells per μl (reference range, 471–1,220), and 200 CD8^+^ T cells per μl (reference range, 303–1,003) in blood. The first lumbar puncture in our hospital revealed the opening pressure was 150 mmH_2_O, with a microprotein level of 11.8 g/L (reference range, 0.15–0.45 g/L), a chloride ion level of 108 mmol/L (reference range, 120–130 mmol/L), GLU was 5.04 mmol/L (reference range, 2.5–4.4 mmol/L). There were 10 nucleated cells per microliter (Table 1). The six subsequent lumbar punctures performed at our hospital revealed a high opening pressure and mononuclear-predominant CSF pleocytosis, hyperglycorrhachia, a high level of protein, and a low level of chloride ion. Nevertheless, the acid-fast staining, mycobacterium cultures, Xpert MTB and RT-PCRs of all CSF samples were negative (Table 1). Enhanced MRI of the brain and cervicothoracic region revealed abnormal enhanced meningeal pia and subarachnoid spaces with signs of communicating hydrocephalus (Figures 1, 2). Enhanced MRI of the lumbar spine (performed on 17 February 2022) revealed nodules and irregularly thickened meninges (Figure 3).

**Figure 4:**
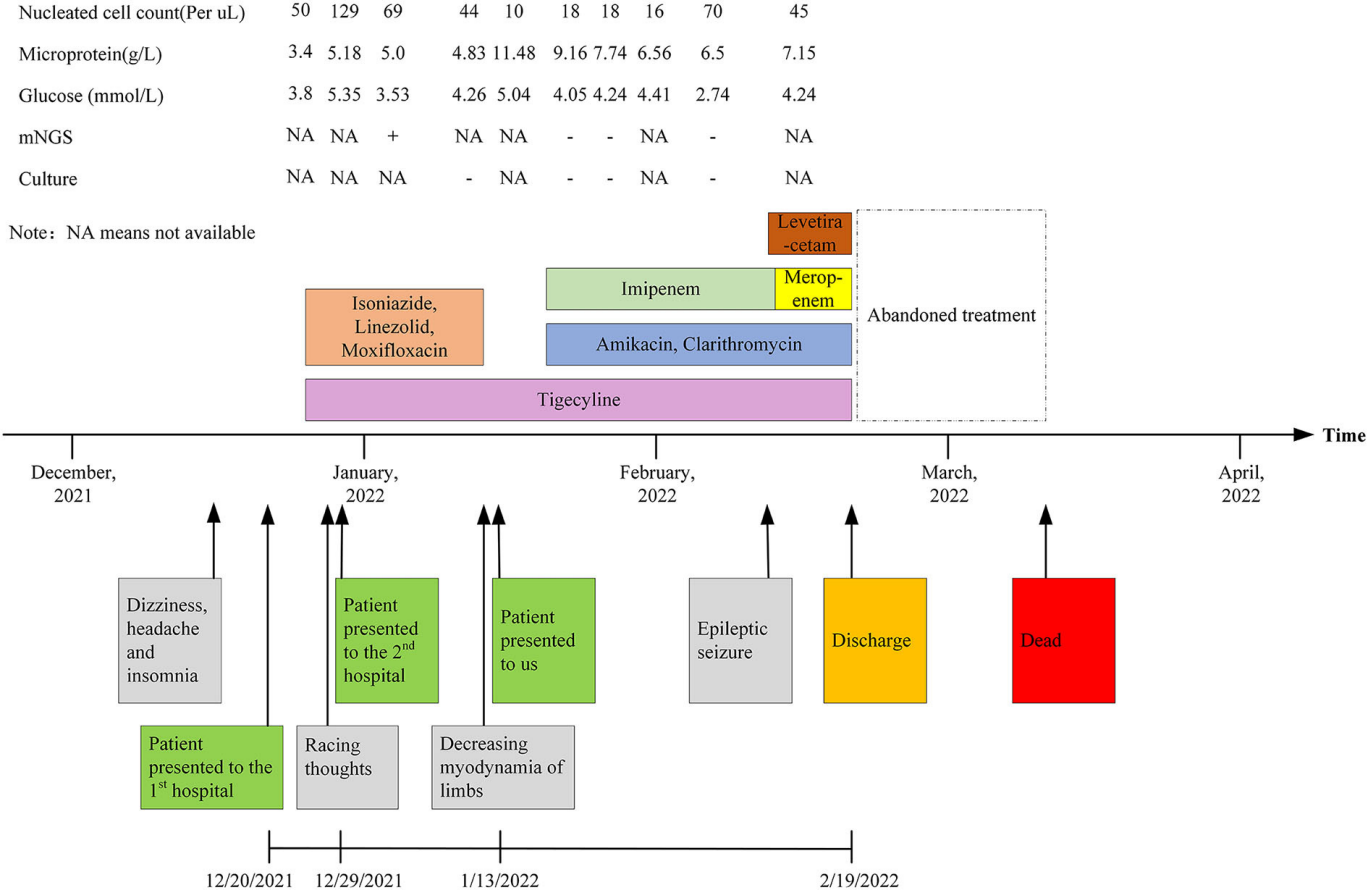
Clinical course, CSF findings, and antibiotic regimens for our case.

It is difficult to diagnose infections caused by NTM since these diseases are rare and their clinical manifestations lack specificity. Most patient with NTM-CNS has been diagnosed with an immunocompromised disease, such as HIV. Meanwhile, it is not helpful to distinguish nervous system infections caused by NTM from other infection types, such as tuberculous infections, on radiological images. Some NTM-CNS infections could present with hydrocephalus or brain atrophy, which is unexplained and not age-related (6, 7), radiological images may also show nodular basal enhancement in NTM-CNS infection (8). On the basis of these radiological and clinical manifestations, as well as the mNGS CSF result in the second hospital, this patient was considered to have a CNS infection caused by *M. houstonense*, which is an NTM. According to the Chinese NTM guidelines (9), amikacin, tigecycline, clarithromycin, and imipenem were administered (Figure 4). After 25 days of therapy, the patient still had intracranial hypertension (CSF pressure > 300 mmH_2_O, the number of nucleated cells in the CSF ranged from 10 to 70/μl, and vision was impaired), and epileptic seizure occurred once. Therefore, we placed continuous lumbar cisterns to drain ~200 ml/day, and the draining CSF was slightly flocculent. Levetiracetam was also administered. Imipenem was then replaced with meropenem on the 27th hospital day.

The patient's CSF culture and mNGS (performed three times in the Precision Medicine Center of our hospital) remained negative throughout the course of therapy in our hospital. The patient did not improve despite appropriate empirical treatment. Finally, the patient lost confidence and requested discharge. The patient still had poor visual acuity, intermittent headaches, and vomiting, and his condition gradually worsened after discharge. Unfortunately, he died 19 days after discharge.

## Discussion

To the best of our knowledge, this is the first report to reveal a CNS infection caused by *M. houstonense* in China. Cases of humans infected with *M. houstonense* are rare throughout the world, and the mechanism of infection with this organism is unclear. Some studies have revealed that freshwater fish and other fish products, especially retail frozen fish, might be a source of NTM for humans (10). However, in this case, the patient was not an aquaculture worker and was not recently exposed to fish products.

The first report of *M. houstonense* infection in a human in the world was from the USA, the organism was found in a wound on the patient's face (1). In China, there were two reported cases of *M. houstonense* infection in humans, one was led to endophthalmitis and the other led to surgical wound infection following an open humeral fracture (11, 12). As for the respiratory system, one case report showed that infection with *M. houstonense* and *M. senegalense* would lead to greater difficulties in the nursing of patients with chronic moderate persistent asthma. According to the current works of literature, human infections induced by *M. houstonense* has mostly been reported as soft tissue infection in humans; CNS infection with RGM is rare. Most reported cases were caused by *M. fortuitum* in the *M. fortuitum* group, and meningitis mostly occurred after invasive operations or trauma (8, 13). This is the first study to report a CNS infection caused by *M. houstonense* without any suspected causes of infection, suggesting a new site of *M. houstonense* invasion.

As mentioned above, *M. houstonense* is an acid-fast, gram-positive, pleomorphic bacilli, and must be cultured in blood agar at 35°C for more than 2 days (1). In this case, the acid-fast staining, mycobacterium cultures, Xpert MTB, and RT-PCRs of all CSF samples, including the cultures of *M. houstonense*, were negative. *M. houstonense* was detected in the first mNGS. However, the following three times' mNGS were negative, the possible reason for this may be the first mNGS was tested in the initial phase of this disease and the following three time's results of mNGS may be affected by the follow-up treatment. As CSF is a sterile body fluid with a low bacterial load in an infected state, the results of conventional microbiological CSF assays may be negative (14, 15). Therefore, mNGS is a potential approach to diagnose infectious diseases that does not rely on a priori selection, because potential pathogens (viral, bacterial, fungal, and parasitic) can be identified by a single assay (16–18). In one study comparing the sensitivity of mNGS to other tests for diagnosing tuberculous meningitis, mNGS possessed the highest sensitivity (84.44%), followed by Xpert MTB /RIF (40%), RT-PCR (24.44%), MGIT960 culture (22.22%), and AFB smear (0%) (19). Another study found that mNGS detected the pathogen in all five prospectively collected body fluids from patients with potential infection, but the testing results of all conventional microbiological assays (including culture) were negative (20). CNS infection caused by Mycobacteria is a paucibacillary infection, meaning that low numbers of bacilli are needed to cause infection (21), *M. houstonense* is a rare infectious disease in humans, especially in the CNS, and it is entirely possible to get negative results on conventional CSF tests. mNGS has a high potential value in accurate diagnosis, thus, we considered that *M. houstonense* is the pathogen that caused the CNS infection of our patient.

The patient in our case was a 26-year-old man without any underlying diseases (such as HIV, malignancy, or hematological system diseases) or a history of immunosuppressive therapy. However, there were 502 CD3^+^ T cells/μl (reference range, 941–2,226), 269 CD4^+^ T cells/μl (reference range, 471–1,220), and 200 CD8^+^ T cells per microliter (reference range, 303–1,003) in the blood which showed an abnormal immune system. The reason why this young patient gets *M. houstonense* might be the following three reasons. Firstly, in China, the highest and lowest prevalence of NTM infections was 8.6% (7.1–10.5%) and 2.7% (2.1–3.4%) in southeastern and northeastern regions. The prevalence of NTM infections in Sichuan province was 7.7%, which was the third highest in China. Moreover, RGM constituted the major fraction in Southern China in contrast to Northern China (44.1 vs. 21.9%) (22). Our patient is a resident of Sichuan province, which is located in Southern China. Secondly, NTM disease is associated with suppressed T-cell-mediated response. Previous studies suggested that patients with NTM disease have lower counts of CD4^+^ T cells, as well as a higher apoptosis rate on CD4^+^ lymphocytes compared to healthy donors (23, 24). NTM are opportunistic human pathogens that colonize macrophages (25). Our patient has a severe decrease in CD4^+^ T cells (<300 cells/μl), which would increase the risk of opportunistic infections and decrease the production of interferon (IFN)-γ, a macrophage activator produced by T cells or natural killer cells that could enhance the cooperation between dendritic cells and CD4^+^ T cells (26–29). Thirdly, some researchers have recently suggested that IFN-γ autoantibodies (AIGAs) may be a new form of late-onset immunodeficiency leading to severe mycobacterial infections (30). Patients infected by NTM commonly express high levels of AIGAs despite being previously healthy and mostly HIV negative, but they did not respond well to antibiotics (31, 32). A study from Thailand suggested that autoimmune disease caused by AIGAs is a major risk factor for extrapulmonary NTM infections (33). The presence of AIGAs might be one of the reasons why the patient was infected with *M. houstonense*. Unfortunately, the patient's family refused the test for AIGAs due to the shortage of cost. Therefore, according to the immune status (decreased CD4^+^ T cells), the result of the first mNGS, and the resident of this patient, the patient got CNS infection caused by *M. houstonense*.

There are still large gaps in antimicrobial therapies for *M. houstonense* infections, and more research is needed to develop a standardized therapeutic regimen for *M. houstonense*. Some studies have suggested that for serious diseases caused by the *M. fortuitum* group, the aminoglycoside amikacin combined with a β-lactam (cefoxitin or imipenem, and imipenem is the most potent member of β-lactam according to current researches), inhibits 100% of *M. fortuitum* isolates (34). Another study found that the *M. fortuitum* group presented with >90% susceptibility to amikacin, cefoxitin, ciprofloxacin, gatifloxacin, imipenem, levofloxacin, linezolid, sulfamethoxazole or trimethoprim-sulfamethoxazole, as well as <90% susceptibility to clarithromycin, doxycycline, and vancomycin (13). Therefore, amikacin, cefoxitin, imipenem, sulfamethoxazole, and fluoroquinolones are usually recommended for *M. fortuitum* group (34). Some studies found that isolates of the unnamed third biovariant complex of the *Mycobacterium fortuitum* group were resistant to doxycycline and one-third were resistant to cefoxitin, all were susceptible to amikacin, ciprofloxacin, sulfamethoxazole, tigecycline, and imipenem also presented with >90% susceptibility to linezolid *in vitro*, the potential molecular mechanisms of linezolid resistance involved the presence of efflux pumps, and mutations in genes encoding for 23S rRNA and ribosomal proteins (35–38). Previous studies have shown tigecycline to be highly active (MIC ≤ 0.12 μg/ml) against the unnamed third biovariant complex of the *Mycobacterium fortuitum* group (sorbitol-positive) (39), and indicated that it could be a potential therapy to treat *M. houstonense*. One study suggested that only 9% of the *M. fortuitum* third biovariant (sorbitol-positive) is susceptible to clarithromycin (MIC, ≤ 4 μg/ml), however, the study did not indicate the specific strain. As for the potential clarithromycin resistance of *M. houstonense*, a possible mechanism could be the presence of erm genes. However, the data and clinical tests to support this conclusion are limited, and clarithromycin is still the cornerstone of management for NTM infectious diseases (40–42). Different sites and populations of *M. houstonense* infection may have different antimicrobial susceptibilities. A previous case report described an old man with surgical wound infection caused by *M. houstonense* that showed antimicrobial susceptibility of resistance to clarithromycin (11), however, it is not clear whether central *M. houstonense* infection is resistant to clarithromycin due to the negative culture. In this case, the treatment is based on drugs recommended in the Chinese NTM treatment guidelines, which didn't provide specific guidance for different strains (9).

This patient's condition did not improve after treatment with amikacin, tigecycline, clarithromycin, and imipenem(meropenem). We consider that one of the reasons for this might be inadequate treatment, as the patient was discharged after 1 month of treatment. The NTM guidelines state that the minimal treatment course is 4 months for skin and soft tissue infection, and 6 months for bone infection (9). A study of CNS infections caused by RGM showed that among survivors, the duration of treatment varies from 2.5 months to more than 16 months (8). Therefore, an inadequate treatment course might be the reason for the poor prognosis. Another reason may be the intracranial infection caused by *M. houstonense*, which is unknown to the medical field currently ease.

## Conclusion

This is the first time that *M. houstonense* has been identified in CSF and has shown a poor prognosis after NTM treatments. Therefore, clinicians should pay more attention to CNS infections caused by the *M. fortuitum* group, more research is needed to explore the treatment for this group of infections, especially for *M. houstonense*.

## Data availability statement

The raw data supporting the conclusions of this article will be made available by the authors, without undue reservation.

## Ethics statement

The treatment for the patient is performed under the tenets of the Declaration of Helsinki. The patient reported in the study provided written informed consent for the treatment. Written informed consent was obtained from the patient for the publication of any potentially identifiable images or data included in this article.

## Author contributions

LW drafted the manuscript. FW helped and revised the manuscript. CY was in charge of the data collection. All authors read and approved the final manuscript.

## Funding

This work was funded by 1.3.5 Project for Disciplines of Excellence, West China Hospital, Sichuan University (ZYJC18021), Sichuan Province Science and Technology Support Program (No. 2021YFQ0030), National Natural Science Foundation of China (No. 82100075), and Post-doctor Research Project, West China Hospital (2021HXBH074).

## Conflict of interest

The authors declare that the research was conducted in the absence of any commercial or financial relationships that could be construed as a potential conflict of interest.

## Publisher's note

All claims expressed in this article are solely those of the authors and do not necessarily represent those of their affiliated organizations, or those of the publisher, the editors and the reviewers. Any product that may be evaluated in this article, or claim that may be made by its manufacturer, is not guaranteed or endorsed by the publisher.
