# Supplementary material for: Central nervous system infection caused by Mycobacterium houstonense: A case report
Source: Front Neurol. 2022 Sep 1;13:908086. doi: 10.3389/fneur.2022.908086 (PMC9475202; doi:10.3389/fneur.2022.908086)
Supplement: Supplementary file 1 [file Data_Sheet_1.PDF]

中文名	拉丁名	序列数
未检出		

非结核分枝杆菌（复合群）

中文名	拉丁名	序列数
未检出		

支原体/衣原体

属				种		
中文名	拉丁名	序列数	相对丰度	中文名	拉丁名	序列数
未检出						

补充列表[e]

类型	属				种		
	中文名	拉丁名	序列数	相对丰度	中文名	拉丁名	序列数
G⁺细菌	葡萄球菌属	*Staphylococcus*	23	0.57%	表皮葡萄球菌	*Staphylococcus epidermidis*	13
病毒	-	-	-	-	人类疱疹病毒7型	Human betaherpesvirus 7	1
G⁺细菌	分枝杆菌属	*Mycolicibacterium*	264	100.00%	休斯顿分枝杆菌	*Mycolicibacterium houstonense*	264

number of gene sequences

参数说明:

类型[a]：G^+：革兰氏阳性菌；G^-：革兰氏阴性菌。

拉丁名[b]：微生物分类学意义上的对本物种的国际通用正式命名。

序列数[c]：指在属/种水平上匹配到该微生物病原体的序列数目。

相对丰度[d]：该微生物在整个标本中检测到的相同类型微生物中所占的比重。

补充列表[e]：指排除了实验室污染后所检测出的、具有致病可能的微生物。其包括样本采集或分装过程中引入的、具有可能致病性的环境机会致病菌或其核酸；来自患者及样本采集人员的人体共生条件致病菌或其核酸；以及定植于病人特定身体部位的微生物。
